# Physical Activity, Heart Rate Variability, and Ventricular Arrhythmia During the COVID-19 Lockdown: Retrospective Cohort Study

**DOI:** 10.2196/51399

**Published:** 2024-02-05

**Authors:** Sikander Z Texiwala, Russell J de Souza, Suzette Turner, Sheldon M Singh

**Affiliations:** 1 Schulich Heart Center Sunnybrook Health Sciences Toronto, ON Canada; 2 Department of Health Research Methods, Evidence, and Impact Faculty of Health Sciences, McMaster University Hamilton, ON Canada; 3 Population Health Research Institute Hamilton Health Sciences Corporation Hamilton, ON Canada; 4 Lawrence Bloomberg Faculty of Nursing University of Toronto, ON Canada; 5 Temerty Faculty of Medicine University of Toronto Toronto, ON Canada

**Keywords:** implantable cardioverter defibrillator, heart rate variability, physical activity, lockdown, ICD, ventricular arrhythmias, defibrillator, implementation

## Abstract

**Background:**

Ventricular arrhythmias (VAs) increase with stress and national disasters. Prior research has reported that VA did not increase during the onset of the COVID-19 lockdown in March 2020, and the mechanism for this is unknown.

**Objective:**

This study aimed to report the presence of VA and changes in 2 factors associated with VA (physical activity and heart rate variability [HRV]) at the onset of COVID-19 lockdown measures in Ontario, Canada.

**Methods:**

Patients with implantable cardioverter defibrillator (ICD) followed at a regional cardiac center in Ontario, Canada with data available for both HRV and physical activity between March 1 and 31, 2020, were included. HRV, physical activity, and the presence of VA were determined during the pre- (March 1-10, 2020) and immediate postlockdown (March 11-31) period. When available, these data were determined for the same period in 2019.

**Results:**

In total, 68 patients had complete data for 2020, and 40 patients had complete data for 2019. Three (7.5%) patients had VA in March 2019, whereas none had VA in March 2020 (*P*=.048). Physical activity was reduced during the postlockdown period (mean 2.3, SD 1.6 hours vs mean 2.1, SD 1.6 hours; *P*=.003). HRV was unchanged during the pre- and postlockdown period (mean 91, SD 30 ms vs mean 92, SD 28 ms; *P*=.84).

**Conclusions:**

VA was infrequent during the COVID-19 pandemic. A reduction in physical activity with lockdown maneuvers may explain this observation.

## Introduction

Increased ventricular arrhythmias (VAs) have been reported with acts of terror and environmental disasters [1,2]. The onset of the COVID-19 pandemic was associated with increased levels of stress [3]. Although one may have anticipated an increased rate of VA during this time, this was not borne in North America and Europe [4,5]. A mechanism to explain this has not been elucidated.

Patients with implantable cardioverter defibrillators (ICDs) are at risk for VA. ICDs contain sensors that can quantify the physical activity of a patient who has an ICD implanted. Acute increases in physical activity can increase the risk of VA [6]. ICDs monitor the changes in the patient’s heart rate. Heart rate variability (HRV) summarizes the beat-to-beat changes in heart rate and reflects the balance between the sympathetic and parasympathetic nervous system. A reduction in HRV can occur during times of stress due to increased sympathetic activation. A reduction in HRV predicts VA [7]. Assessing the changes in physical activity and HRV may provide insight into the lack of increased VA observed during the onset of the COVID-19 lockdown.

Herein, we report the changes in physical activity and HRV in patients with ICD during the COVID-19 lockdown of March 2020 in Ontario, Canada.

## Methods

### Study Cohort

Sunnybrook Hospital is a regional cardiac center in Ontario, Canada. The Sunnybrook ICD clinical electronic medical record database (Paceart Optima System, version 1.8.269.0; Medtronic) was searched to identify all actively followed patients with ICD with data on physical activity and HRV in March 2020. As this was a retrospective observational study, patients were not actively recruited rather all patients with the available data were included in this retrospective cohort study.

### Study Periods

Our study focused on the first month of the COVID-19 pandemic (March 2020). March 1-10, 2020, was designated as the prelockdown period and March 11-31, 2020, as the lockdown period. During the latter period, the World Health Organization declared the COVID-19 outbreak a pandemic (March 11), with a subsequent crash in North American stock markets (March 12) and a declaration of a state of emergency in the United States (March 13) and Ontario (March 17).

### VA, Physical Activity, and Heart Rate Variability

ICDs record all VA. The presence of VA requiring an ICD therapy during the study period was documented. Patient physical activity is recorded when a patient moves at a rate above a minimum threshold of 2 miles per hour. ICDs quantify the amount of time spent moving above this rate.

ICD algorithms determine HRV as the SD of the average sinus intervals over 5 minutes, averaged over 24 hours (288 periods). This time domain approach to determine HRV provides the best prognostic information [7]. As the knowledge of atrial activity is necessary to determine the HRV, it cannot be determined in patients who do not have a dual chamber ICD (ie, a device with an atrial lead). Furthermore, HRV cannot be determined in the presence of inherently irregular arrhythmia such as atrial fibrillation. As such, patients with a single chamber ICD and a history of atrial fibrillation were excluded from the study.

Physical activity and HRV during the study period were extracted using an open-source software tool (WebPlotDigitizer, version 4.4). To provide an estimate of the change in physical activity and HRV between the 2 study periods, the extracted daily values for physical activity and HRV were averaged over the pre- and lockdown periods. Where available, VA, physical activity, and HRV were obtained from March 1 to 31, 2019, to act as a control.

### Statistical Analysis

Participant characteristics are presented as mean (SD) or counts (%). Chi-square testing was used to determine the differences in the percentage of patients experiencing VA during the study periods. Two-tailed paired *t* tests and analysis of covariance were used to compare HRV and physical activity between the 2 study periods.

Statistical analyses were performed using the statistical analysis system statistical software package (version 9.4; SAS Institute Inc). *P* values <.05 were considered statistically significant.

### Ethical Considerations

The Sunnybrook Hospital research ethics board approved the study (study 1632). The requirement for patient consent was waived by the research ethics board. Data were collected in anonymously. There was no patient compensation for participation in this study.

## Results

Of the 650 actively followed ICD patients, 485 did not have data on both physical activity and HRV, and 97 patients did not have follow-up during the COVID-19 pandemic. The final cohort comprised of 68 patients, 40 of whom had HRV and physical activity data for 2021 and 2020.

The average age of the cohort was 70 (SD 11) years and predominantly male (n=52, 77%). Half of the participants (n=34, 50%) had coronary artery disease, 40% (n=27) had ventricular tachycardia, and 37% (n=25) had a cardiac resynchronization device. Beta-blockers and antiarrhythmic drugs were used by 77% (n=52) and 24% (n=16) of the cohort, respectively.

No patient had a VA during the 2020 study period. Three (7.5%) patients experienced VA in 2019, all between March 11 and 30. Thus, there were fewer VA events in the lockdown period of 2020 compared to the equivalent time in 2019 (n=0, 0% vs n=3, 7.5%; *P*=.048).

Activity was reduced by approximately 12 minutes during the lockdown period of 2020 compared to the prelockdown period of 2020 (mean 2.1, SD 1.6 hours vs mean 2.3, SD 1.6 hours; *P*=.003). There was no difference in the average activity in the prelockdown period in 2020 compared to the equivalent dates in 2019 (mean 2.3, SD 1.6 hours vs mean 2.5, SD 1.7 hours; *P*=.06).

HRV was unchanged between the 2020 prelockdown and lockdown periods (mean 91, SD 30 ms vs mean 92, SD 28 ms; *P*=.84). HRV was similar in 2020 and 2019 (prelockdown: mean 89, SD 28 ms vs mean 90, SD 30 ms; *P*=.70 and lockdown: mean 86, SD 26 ms vs mean 90, SD 25 ms; *P*=.30).

## Discussion

### Principal Findings

This work supports prior publications highlighting a lack of increased VA with the onset of the COVID-19 pandemic. Our research is hypothesis generating, which provides a possible mechanism to explain the lack of increased VA observed at the onset of the COVID-19 pandemic. It is speculated that a reduction in physical activity with lockdown maneuvers may have reduced the frequency of VA.

Lockdown maneuvers resulted in stay-at-home orders, closing of gyms or shopping centers, and working from home. These maneuvers, which were similar in Ontario and other jurisdictions, effectively reduced the physical activity of all individuals during this time period. Although small (~12 minutes), it is possible that the reduction in physical activity may have played a role in mitigating arrhythmic risk. For context, a reduction of 12 minutes is at a minimum equivalent to a reduction in walking 0.4 miles or 1000 steps a day. As we are not able to quantify the intensity of the activity, it is possible this reduction in activity could have been higher.

HRV is a marker of autonomic tone and a predictor of VA. We observed no clinically important or statistically significant change in HRV. This finding seems counterintuitive, given the reports of increased distress with the onset of the lockdown maneuvers [3]. We hypothesize that a number of factors may have mitigated additional reductions in HRV in this population. First, the use of beta-blockers was high (n=52, 77%) in this population. Prior work has demonstrated that beta-blockers can preserve autonomic balance in the setting of mentally stressful events [8]. Second, patients with ICD already have a high level of circulating catecholamines (evidenced by the depressed HRV even prior to the COVID-19 pandemic). The additional influence of external psychological stresses with the COVID-19 pandemic may not impact overall autonomic tone. Finally, we speculate that, unlike singular unexpected catastrophic events [8], the anticipation of lockdown measures may have lessened this psychological stress. This finding highlights the variable impact of different catastrophic events on the risk of VA.

Limitations to this work include the fact the data were derived from a single center with a relatively small number of patients. Second, the large number of exclusions may have resulted in a highly selected population limiting the applicability to other populations. Third, we limited our assessment to the early part of the pandemic, given the homogenous lockdown interventions and limited impact of lack of access to care during this early time. It is unclear whether these findings would persist into different waves of the pandemic. Finally, the findings were from Ontario, Canada, and may not apply to other jurisdictions with more severe COVID-19 outbreaks and interventions. The primary strength of this work is the precise measure of VA, physical activity, and HRV.

### Conclusions

Physical activity was reduced in patients with ICD during the COVID-19 lockdown. Our observations may provide a possible mechanistic insight into lack of increased VA in patients with ICD during the COVID-19 pandemic. We suggest future work in larger patient populations and other jurisdictions to confirm our findings. Given the long-term benefits of physical activity, we also suggest future work by public health agencies to ensure the observed decline in physical activity at the onset of the COVID-19 pandemic is not sustained.

## Abbreviations

HRV: heart rate variability
ICD: implantable cardioverter defibrillator
VA: ventricular arrhythmia

## Data Availability

The data sets generated and analyzed during this study are available from the corresponding author upon reasonable request.
